# Socioeconomic determinants of teenage pregnancy and early motherhood in the United Kingdom: A perspective

**DOI:** 10.34172/hpp.2021.52

**Published:** 2021-12-19

**Authors:** David Aluga, Elvis Anyaehiechukwu Okolie

**Affiliations:** School of Health and Life Sciences, Teesside University Middlesbrough, United Kingdom

**Keywords:** Pregnancy in adolescence, Mothers, Parturition, Socioeconomic factors, United Kingdom

## Abstract

The United Kingdom has one of the highest teenage birth rates among countries in western Europe. Government initiatives such as the Teenage Pregnancy Strategy introduced by the labor government in 1999 to reduce the teenage pregnancy rate by half in ten years could be responsible for the steady decline in teenage conception and childbirth for the past two decades. However, to sustain this decrease it is crucial to consider the broader socioeconomic and environmental determinants of teenage pregnancy at the population level. A selected literature search was conducted in this respect to highlight the factors that could be neglected by recent interventions on teenage pregnancy and childbirth in the United Kingdom.

## Introduction

Adolescent or teenage pregnancy which often refers to conception in a girl between the ages of 10-19 years^1^ was not regarded as a public health issue in the United Kingdom until the second half of the 20^th^ century.^2,3^ Before this period, it was not uncommon for girls to get married in their late adolescent ages (15-19 years). According to the Office for National Statistics (ONS),^4^ the United Kingdom has the highest rate of teenage births among countries in western Europe. This is despite the implementation of several interventions such as sex education and promotion of sexual health services and highlights the inadequacy of the traditional approaches to controlling teenage conception and birth. High teenage childbearing may be indicative of persistent underlying social problems not solved by existing early childhood and youth programs.^5^ Kearney and Levine^6^ argue that the promotion of abstinence and the use of contraceptives would not be sufficient without correcting the underlying structural and economic problems. Teenage pregnancy has been associated with socioeconomic deprivation resulting in varying education and employment aspirations among young people.^7-11^ Moreover, maternal conditions are still the leading cause of death among adolescent girls aged 15-19 years.^12^ This article intends to explore the numerous socioeconomic and environmental factors affecting adolescent pregnancy and childbirth that could be neglected by current programs.

## Geographical variations in teenage childbearing

Geographical variations in the burden of teenage pregnancies have been observed. In England, the teenage conception rates seem to follow the north-south divide. The most deprived areas in the northeast have high conception but lower abortion rates while the affluent areas of the southeast have low conception but higher abortion rates.^10^ Therefore, teenage pregnancy would be better controlled by economic incentives than restrictive abortion policies.^13^ The poverty due to living in an unequal or marginalized society can cause despair and desperation which could result in choices that lead to immediate gratifications. An adverse circumstance could make a teenage girl consider childbearing as an escape. This outcome might even be more certain when societal beliefs reinforce such expectations.^14^

The choice to opt-in for abortion could be influenced by family socioeconomic status, societal beliefs, and availability of abortion services.^15^ There can also be a strong correlation between income disparities and teenage birth rates with highly unequal populations having higher teenage birth rates.^8,11,16^ Nevertheless, it is improper to conclude that every teenage girl living in a low socioeconomic area will conceive at an early age. The several factors affecting teenage pregnancy and childbirth could be nuanced and difficult to pinpoint which is more relevant in a complex interplay of more than one. There might be important relationships between teenage pregnancy and factors such as parental separation or divorce, sexual, physical, emotional or substance abuse, undiagnosed depression/anxiety, and dislike of school.^17^

## Spotlight on interventions directed at teenage pregnancy in the UK

In 1999, the UK labor government launched a 10-year teenage pregnancy strategy for England aimed at halving conceptions for women under-18. The policy has contributed significantly to the decline in teenage pregnancy. The under-18 pregnancy rate in England and Wales had decreased from a mean of 45.1 per 1000 to a mean of 16.8 per 1000 among girls aged 15-17 years from 1999 to 2018.^10^ However, the percentage of under-18 conceptions that led to abortion increased from 46.5% in 1999 to over 50% in 2018.^10,18^ Sociocultural norms and availability of abortion services could explain the high abortion rates in affluents areas.^19^ Until the issues related to family poverty, low educational attainment and future aspirations are addressed, it might be impossible to sustain the decrease in teenage conceptions and childbirths in deprived areas.^20^

In addition, the teenage pregnancy strategy integrated both social and clinical programs such as awareness campaigns, sexual health services, and sex education in schools. There was also an increase in the number of teenage mothers engaged in employment, education, and training.^21-23^ The low rates of teenage pregnancy and childbirth in the Netherlands and Scandinavian countries have been associated with wider contraceptive use, good sex education, and cultures that promote free and open discussion of sexual matters.^21^ In the United Kingdom, there is still poor use of contraceptives among sexually active adolescents.

## Environmental perception of risks

The teenage girl’s perception of risks in her immediate environment could accurately predict sexual behavior than socioeconomic indices.^24^ Beyond the observed geographical variations in teenage pregnancy in the United Kingdom, individual circumstances could influence early motherhood. Teenage girls who grow up in an unsafe and unpredictable environment might have negative future aspirations which would make them take risky decisions.^25^ On the other hand, teenage girls who had experienced their friend or acquaintance get pregnant at an early age may learn indirectly. They could alter their preferences about early childbirth and decrease their sexual drive by focusing on other aspects of their lives such as their education and career.^26^ Teenage girls who had experienced sexual molestation at early ages could become sexually active at these ages. Low aspirations could be a result of a lack of self-esteem which can cause a teenage girl who is unable to resist peer pressure and sexual abuse to dismiss the use of contraceptives. Education can improve a teenage girl’s self-confidence and delay the age of first intercourse. But a culture that encourages gender power imbalance could lead to secrecy in discussing sexual matters.^27^ In such circumstances, gender inequality becomes a contributing factor to health inequality.^28^ Moreover, the lack of confidentiality in the delivery of sexual health services would hinder teenage girls from seeking such services even when they need them.

## Intergenerational dimension of teenage childbirth

An intergenerational cycle could be at work in teenage pregnancy.^29^ This may be because the common risk factors attributable to teenage pregnancy are often higher among children born to teenage mothers. Kearney and Levine^6^ observe that female children born by a single mother, a teenage mother, or a mother with low educational and economic status can be at higher risk of being pregnant at their teenage ages compared to those without these ‘disadvantages’. The socioeconomic deprivations coupled with the absence of a father figure could make female children born to teenage mothers vulnerable in search of a better life and male companionship. Teenagers can make the decision to get pregnant at an early stage based on their past experiences and expectation of what the future holds for them, and a way to change their perception is to change the environment.^3^ Targeted intervention to this ‘at-risk’ group could be beneficial in breaking the intergenerational cycle that may be present.

Societal norms and values can highly influence the behavior of teenagers and therefore it would be beneficial to adopt a life-course approach to the management of teenage pregnancy.^30,31^ In some communities, teenage mothers are often seen as deviant from the norm and stigmatized by both the public and the media.^32^ These stereotypes could make them adopt different defensive mechanisms to protect themselves and their children, such as socially isolating. Current interventions directed at individual behavioral change may need to adjust to accommodate for broader population-level problems because the individual-level effect may not always translate to population-level impact.^33^

## Conclusion

In conclusion, teenage pregnancy and childbirth may be dependent on a myriad of social, structural, economical, and environmental factors. Current and future interventions should take cognizance of these determinants of teenage pregnancy to maximize their outcomes and address the intergenerational and geographical dimensions. Finally, teenage girls must be deliberately engaged in the design and implementation of interventions targeting early pregnancy and childbearing.

## Funding

None to be declared.

## Competing interests

None to be declared.

## Ethical approval

Not applicable.

## Authors’ contributions

DA was responsible for the conceptualization and writing of the manuscript, and also served as the corresponding author. EAO reviewed and edited the initial draft of the paper before submission. All authors reviewed and accepted the final version of the article.
